# Safety and Efficacy Results of a Phase I, Open-Label Study of Concurrent and Delayed Nivolumab in Combination With *nab*-Paclitaxel and Carboplatin in Advanced Non-small Cell Lung Cancer

**DOI:** 10.3389/fonc.2019.01256

**Published:** 2019-11-26

**Authors:** Jonathan W. Goldman, David M. Waterhouse, Ben George, Peter J. O'Dwyer, Rafia Bhore, Sibabrata Banerjee, Larry Lyons, Chrystal U. Louis, Teng Jin Ong, Karen Kelly

**Affiliations:** ^1^David Geffen School of Medicine at the University of California, Los Angeles, Los Angeles, CA, United States; ^2^Oncology Hematology Care, Cincinnati, OH, United States; ^3^Froedtert & the Medical College of Wisconsin, Milwaukee, WI, United States; ^4^Abramson Cancer Center, University of Pennsylvania, Philadelphia, PA, United States; ^5^Celgene Corporation, Summit, NJ, United States; ^6^Comprehensive Cancer Center, University of California, Davis, Sacramento, CA, United States

**Keywords:** non-small cell lung cancer, *nab*-paclitaxel, carboplatin, nivolumab, treatment beyond progression

## Abstract

**Introduction:** Multicenter, phase I study of concurrent and delayed nivolumab plus *nab*-paclitaxel/carboplatin in advanced non-small cell lung cancer (NSCLC).

**Methods:** Chemotherapy-naive patients with advanced NSCLC (ineligible for potentially curative radiation or surgery) received *nab*-paclitaxel 100 mg/m^2^ (days 1, 8, 15) and carboplatin area under the curve 6 (day 1) intravenously every 21 days (first 4 cycles); nivolumab 5 mg/kg was administered intravenously (day 15) beginning in cycle 1 (concurrent) or cycle 3 (delayed) in separate cohorts and continued beyond the 4 chemotherapy cycles. The primary objective was to assess safety. Secondary objectives were to assess tolerability and explore antitumor activity.

**Results:** All 32 patients received chemotherapy; 20 of 22 and 6 of 10 patients also received concurrent or delayed nivolumab, respectively. No dose-limiting toxicities were reported in the concurrent cohort; 1 dose-limiting toxicity was reported in the delayed cohort. In the concurrent cohort, 20 patients (91%) had ≥1 grade 3/4 treatment-emergent adverse event (TEAE), and 7 (32%) discontinued treatment due to TEAEs. In the delayed cohort, all patients had ≥1 grade 3/4 TEAE, and 2 (20%) discontinued due to TEAEs. The median progression-free and overall survival, respectively, were 10.5 and 29.3 months in the concurrent cohort and 4.1 and 8.2 months in the delayed cohort.

**Conclusions:** The safety profile of the combination was consistent with that of individual agents and generally similar in the 2 cohorts. Efficacy outcomes in the concurrent cohort, but not in the delayed cohort, were encouraging and support the rationale for concurrent administration of nivolumab with *nab*-paclitaxel/carboplatin for the treatment of advanced NSCLC.

**Clinical Trial Registration:** www.ClinicalTrials.gov, identifier: NCT02309177

## Introduction

Recent evidence has demonstrated the safety and efficacy of immune checkpoint inhibitors as monotherapy or in combination with chemotherapy for the treatment of non-small cell lung cancer (NSCLC) (1). At the time that the current study was designed, chemotherapy was hypothesized to augment the effects of immune checkpoint inhibitors (2), which might lead to increased antitumor activity and, ultimately, improved survival rates. According to the current National Comprehensive Cancer Network guidelines, treatment regimens consisting of immunotherapy (atezolizumab or pembrolizumab) plus platinum-doublet chemotherapy (plus bevacizumab in the case of atezolizumab for non-squamous histology) are category 1 first-line treatment options for patients with advanced NSCLC, based on the outcomes of the KEYNOTE-189, IMpower150, and KEYNOTE-407 trials (3–6).

The overall safety and efficacy of the *nab*-paclitaxel/carboplatin doublet support its potential as a therapeutic backbone for combination with immune checkpoint inhibitors. The phase III KEYNOTE-407, IMpower130, and IMpower131 trials have demonstrated improved outcomes with *nab*-paclitaxel–based regimens in combination with immunotherapy agents (pembrolizumab or atezolizumab) in patients with metastatic NSCLC (5, 7, 8). Furthermore, *nab*-paclitaxel regimens offer a theoretical benefit in that they do not require corticosteroid premedication, thus avoiding the potential for steroid-induced immune suppression.

The current multicohort, phase I trial evaluated nivolumab combined with *nab*-paclitaxel–based regimens in advanced solid tumors (pancreatic, NSCLC, and breast); the results from the 2 NSCLC cohorts are reported here. At the time that the current study was designed, it was not completely clear whether concurrent administration of immune checkpoint inhibitors with chemotherapy was as safe and efficacious as delayed administration. A phase II trial in advanced NSCLC suggested better efficacy with delayed vs. concurrent administration of ipilimumab with chemotherapy (carboplatin/paclitaxel) (9). However, the efficacy of concurrent vs. delayed administration of an immune checkpoint inhibitor in combination with chemotherapy could depend on the inhibitor's mechanism of action and how it interacts with the cancer-immunity cycle (2). Therefore, the NSCLC cohorts of the current study were designed with nivolumab administration concurrently with *nab*-paclitaxel/carboplatin at cycle 1 or delayed until cycle 3.

## Materials and Methods

### Study Oversight

The study (NCT02309177) was approved by the institutional review board or independent ethics committee at each study site (listed in Supplementary Table 1) and conducted in accordance with Good Clinical Practice, as described in the International Conference on Harmonisation E6 guidelines, and with the ethical principles outlined in the Declaration of Helsinki (10). All patients provided written informed consent before any study procedures were initiated.

A data review committee, comprising investigators and the sponsor, was established to monitor the conduct of the study and to confirm dose level and/or identify the need for dose de-escalation. A safety oversight subcommittee, made up of data review committee members, assessed safety and tolerability in part 1 and identified the recommended part 2 dose (RP2D) to be administered in the expansion phase.

### Study Population

Patients (aged ≥18 years) with histologically or cytologically confirmed stage IIIB or IV NSCLC who had not previously received chemotherapy or investigational therapy for the treatment of metastatic disease and who were not candidates for curative surgery or radiation were eligible. Adjuvant or neoadjuvant chemotherapy was allowed if it was completed >12 months prior to randomization, with no disease progression or recurrence during those 12 months. Patients were required to have measurable disease according to Response Evaluation Criteria in Solid Tumors (RECIST) version 1.1 guidelines and an Eastern Cooperative Oncology Group performance status (ECOG PS) of 0 or 1. Patients who received prior therapy with immune checkpoint inhibitors or had grade ≥2 peripheral neuropathy or any lung disease that could potentially interfere with detection or management of suspected drug-related pulmonary toxicity were excluded.

### Study Design and Treatment

This phase I, open-label, multicenter study evaluated the safety and efficacy of nivolumab plus *nab*-paclitaxel–based regimens in advanced solid tumors in 6 cohorts: 2 each for pancreatic cancer, NSCLC, and metastatic breast cancer. Here, we report the outcomes in the 2 NSCLC cohorts, in which nivolumab initiation was either concurrent with chemotherapy in cycle 1 or delayed to cycle 3.

All patients received *nab*-paclitaxel 100 mg/m^2^ intravenously on days 1, 8, and 15 and carboplatin area under the curve 6 mg/mL/min intravenously on day 1 every 21 days for the first 4 cycles. Based on the available evidence at the time (11–13), nivolumab was dosed at 5 mg/kg intravenously; it was given on day 15 of each 21-day cycle, starting either with *nab*-paclitaxel/carboplatin in cycle 1 (concurrent) or in cycle 3 (delayed). Dosing of nivolumab on day 15 of each cycle was intended to minimize potential adverse events arising from interactions between chemotherapy and nivolumab (i.e., through dose adjustment of *nab*-paclitaxel on day 8, if warranted). Patients continued to receive nivolumab alone beyond 4 cycles. Nivolumab dose was determined based on dose-limiting toxicities (DLTs); once established, the nivolumab dose could not be reduced for individual patients, but it could be modified (see Supplementary Methods for details). Treatment could continue until disease progression (RECIST 1.1), unacceptable toxicity, or withdrawal of consent. If a treatment-related unacceptable toxicity was attributed to only 1 agent in the combination, patients were permitted to remain in the study and receive other agent(s) per the assigned schedule. Patients were permitted to continue nivolumab treatment beyond initial RECIST 1.1–defined progressive disease if they met a set of specific criteria (see Supplementary Methods for details). The study therapy was to be stopped if further disease progression was documented. Supportive care was permitted at the discretion of the investigator.

**Figure 1:**
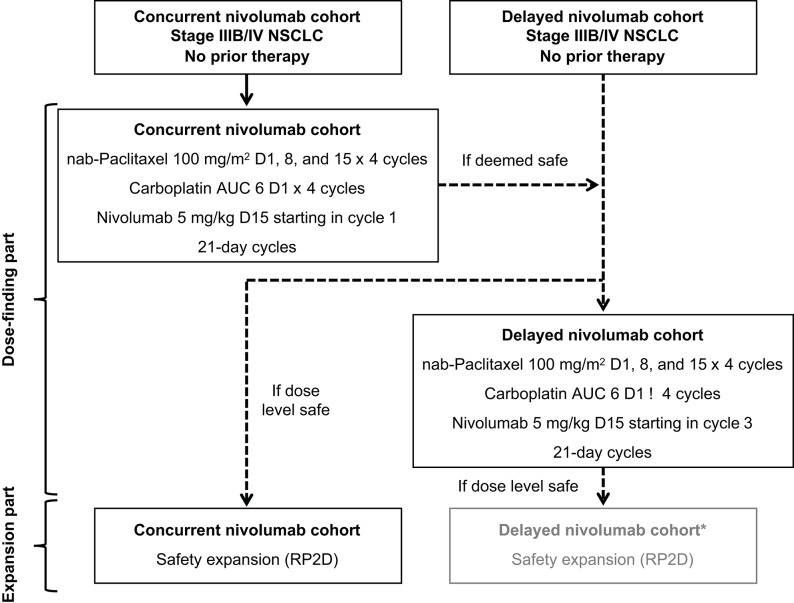
Study design. *The expansion part of the delayed cohort was not pursued because the survival outcomes in the dose-finding part of this cohort were not as encouraging as those in the concurrent cohort and the safety profile with concurrent nivolumab administration was acceptable. AUC, area under the curve; NSCLC, non–small-cell lung cancer; RP2D, recommended part 2 dose.

The study was designed with 2 parts: a dose-finding part and an expansion part (Figure 1). In the dose-finding part, DLTs were to be evaluated. In the expansion part, tolerability was to be further characterized and antitumor activity was to be explored. Patients were initially assigned to the concurrent cohort, and if the dose-finding part was deemed safe, the RP2D was to be declared and patients were to be assigned in a non-randomized manner to either the expansion part of the concurrent cohort or the dose-finding part for the delayed cohort (Figure 1; see Supplementary Methods for additional details).

### Study Endpoints

The primary endpoints of the study were the number of patients with DLTs in the dose-finding part of each treatment cohort (see Supplementary Methods for definitions of DLT and DLT-evaluable population) and the percentage of patients with grade 3/4 treatment-emergent adverse events (TEAEs) or treatment discontinuation due to a TEAE during the study. Secondary endpoints included TEAEs leading to dose reduction, dose delay, or treatment discontinuation; investigator-assessed progression-free survival (PFS); overall survival (OS); disease control rate (DCR); overall response rate (ORR); and duration of response (DOR; all responses were based on RECIST 1.1 criteria. Exploratory endpoints included correlation between programmed death ligand 1 (PD-L1) expression prior to treatment and tumor response.

### PD-L1 Measurement

Expression of PD-L1 in tumor biopsy samples was analyzed using a validated, automated immunohistochemistry assay with the rabbit monoclonal antibody 28-8 pharmDx kit (Agilent, Carpinteria, CA) and Signatera (RUO) kit (Natera, San Carlos, CA). The percentage of tumor cells with complete circumferential or partial linear membrane staining of any intensity, but not cytoplasmic staining, was used to score PD-L1 staining.

### Statistical Analyses

The treated population comprised patients who received ≥1 dose of any investigational product. Separately, analyses were also conducted on the subset of patients who received ≥1 dose of nivolumab. However, because nivolumab treatment in the delayed cohort depended on whether patients stayed on treatment through cycle 3, treatment efficacy in these 2 populations should not be compared.

At least 6 DLT-evaluable patients were needed at the given dose level to determine the RP2D. If a treatment was expanded to part 2, additional patients were enrolled to achieve a total of 20 nivolumab-treated patients. Twenty nivolumab-treated patients allowed accurate assessment of the incidence of adverse events (AEs).

TEAEs were summarized based on the Medical Dictionary for Regulatory Activities (MedDRA) version 21.0 guidelines and graded for severity using the National Cancer Institute's Common Terminology Criteria for Adverse Events version 4.0. The ORR and DCR were reported using proportions and exact 95% confidence intervals (CIs), and the PFS, OS, and DOR were summarized using the Kaplan-Meier method with median times and 95% CIs. Patients who discontinued treatment for reasons other than disease progression, consent withdrawal, or initiation of a new anticancer therapy were followed up for tumor response assessments and new anticancer therapies.

## Results

The results presented here are from the database cutoff date of September 12, 2018, with the median follow-up time for OS (reverse Kaplan-Meier estimates) of 35.9 months for the concurrent cohort and 30.7 months for the delayed cohort.

### Patients and Treatment

Among patients assigned to receive concurrent nivolumab, 22 were treated (20 received concurrent nivolumab) at 8 sites from May 2015 through June 2018: 12 patients were enrolled in the dose-finding part (6 of these were the DLT-evaluable patients), and 10 were enrolled in the expansion part. In this cohort, 2 patients did not receive nivolumab due to AEs. Patients in this cohort discontinued treatment due to progressive disease [11 patients (50.0%)], AEs [6 patients (27.3%)], withdrawal by patient [3 patients (13.6%)], or other reasons [2 patients (9.1%)].

Among patients assigned to receive delayed nivolumab, 10 were treated (6 received delayed nivolumab and were DLT-evaluable) at 7 sites from January 2016 through September 2018 and were enrolled in the dose-finding part. In this cohort, 4 patients discontinued treatment due to progressive disease before cycle 3 and, therefore, did not receive nivolumab. Patients in this cohort discontinued treatment due to progressive disease [7 patients (70.0%)], AEs [2 patients (20.0%)], or study termination [1 patient (10.0%)]. This arm was not expanded.

Demographic and baseline clinical characteristics are reported in Table 1. The median age was 65.0 years in the concurrent cohort and 70.0 years in the delayed cohort; among patients with confirmed histology, adenocarcinoma was most common (54.5 and 50.0%, respectively). Most patients in the concurrent cohort were female (72.2%), had ECOG PS of 1 (68.2%), and had PD-L1 expression ≥1% (54.5%). In the delayed cohort, most patients were male (70.0%), had ECOG PS of 0 (60.0%), and had PD-L1 expression <1% (50.0%) [among 70% with available data] (Table 1). The patients in the nivolumab-treated subset showed similar trends (Supplementary Table 2).

**Table 1:** Demographic and baseline clinical characteristics.

**Characteristic**	**Concurrent cohort (*n* = 22)**	**Delayed cohort (dose-finding part only) (*n* = 10)**
Age, median (range), years	65.0 (38–77)	70.0 (44–82)
<65 years, *n* (%)	10 (45.5)	4 (40)
≥65 years, *n* (%)	12 (54.5)	6 (60)
Sex, *n* (%)		
Male	6 (27.3)	7 (70.0)
Female	16 (72.7)	3 (30.0)
Race, *n* (%)		
White	18 (81.8)	8 (80.0)
Asian	1 (4.5)	0
Black or African American	0	2 (20.0)
Not collected or reported	3 (13.6)	0
ECOG PS, *n* (%)		
0	7 (31.8)	6 (60.0)
1	15 (68.2)	4 (40.0)
Stage at primary diagnosis, *n* (%)		
IA	2 (9.1)	0
IB	1 (4.5)	0
IIB	0	1 (10.0)
IIIA	2 (9.1)	1 (10.0)
IIIB	1 (4.5)	2 (20.0)
IVA	10 (45.5)	3 (30.0)
IVB	4 (18.2)	2 (20.0)
Unknown	2 (9.1)	1 (10.0)
Histology, *n* (%)		
Confirmed	21 (95.5)	10 (100.0)
Adenocarcinoma	12 (54.5)	5 (50.0)
Squamous cell carcinoma	7 (31.8)	2 (20.0)
Large cell carcinoma	0	1 (10.0)
Other	2 (9.1)	2 (20.0)
Not confirmed	1 (4.5)	0
PD-L1 category, *n* (%)		
<1%	6 (27.3)	5 (50.0)
≥1%	12 (54.5)	2 (20.0)
Missing	4 (18.2)	3 (30.0)
*KRAS* status, *n* (%)		
*KRAS* mutant	3 (13.6)	2 (20.0)
*KRAS* wild type	3 (13.6)	2 (20.0)
Unknown	16 (72.7)	6 (60.0)
*ALK* status, *n* (%)		
*ALK* wild type	9 (40.9)	6 (60.0)
Unknown	13 (59.1)	4 (40.0)
*EGFR* status, *n* (%)		
*EGFR* mutant	2 (9.1)	1 (10.0)
*EGFR* wild type	8 (36.4)	4 (40.0)
Unknown	12 (54.5)	5 (50.0)
Prior anticancer therapy, *n* (%)		
Systemic therapy^a^	5 (22.7)	1 (10.0)
Radiation	9 (40.9)	3 (30.0)
Surgery	5 (22.7)	5 (50.0)

*ALK, anaplastic lymphoma kinase; ECOG PS, Eastern Cooperative Oncology Group performance status; EGFR, epidermal growth factor receptor; PD-L1, programmed death ligand 1*.

a *Two patients in the concurrent cohort received a tyrosine kinase inhibitor (1 each received afatinib and erlotinib); none of the patients in the delayed cohort received a tyrosine kinase inhibitor*.

### Safety Outcomes and Treatment Exposure (Concurrent Cohort; Dose-Finding and Expansion Parts)

No DLTs were reported during the dose-finding part in the concurrent cohort. All patients had ≥1 all-grade TEAE; ≥1 grade 3/4 TEAE was reported in 90.9% of patients (Table 2). At least 1 serious TEAE was reported in 36.4% of patients. Grade 3/4 TEAEs that occurred in ≥10% of patients were (MedDRA preferred terms) anemia, neutropenia, neutrophil count decreased, white blood cell count decreased, hypokalemia, and vomiting. Grade 1/2 peripheral neuropathy was observed in 11 patients (50%). Results were generally similar in the nivolumab-treated subset (Supplementary Table 3). At least 1 all-grade or grade 3/4 TEAE of special interest attributable to nivolumab was reported in 16 (80%) and 3 (15%) of nivolumab-treated patients, respectively; grade 1/2 hypothyroidism and pneumonitis occurred in 3 (15.0%) and 2 (10.0%) patients, respectively (Supplementary Table 3).

**Table 2:** Safety outcomes, including most common TEAEs.

**Parameter, *n* (%)**	**Concurrent cohort (*****n*** **=** **22)**		**Delayed cohort (dose-finding part only) (*****n*** **=** **10)**	
	**All grade**	**Grade 3/4**	**All grade**	**Grade 3/4**
Patients with ≥1 TEAE	22 (100.0)	20 (90.9)	10 (100.0)	10 (100.0)
Patients with ≥1 serious TEAE	8 (36.4)	6 (27.3)	3 (30.0)	2 (20.0)
Most common TEAEs^a^				
Vomiting	13 (59.1)	3 (13.6)	3 (30.0)	0
Anemia	13 (59.1)	10 (45.5)	7 (70.0)	4 (40.0)
Neutropenia	11 (50.0)	9 (40.9)	5 (50.0)	5 (50.0)
Thrombocytopenia	8 (36.4)	2 (9.1)	2 (20.0)	1 (10.0)
Neutrophil count decreased	6 (27.3)	6 (27.3)	3 (30.0)	2 (20.0)
Hypokalemia	5 (22.7)	3 (13.6)	1 (10.0)	1 (10.0)
WBC count decreased	5 (22.7)	3 (13.6)	1 (10.0)	0
Pneumonia	3 (13.6)	2 (9.1)	–	–
Hyponatremia	2 (9.1)	2 (9.1)	1 (10.0)	0

*NCI-CTCAE, National Cancer Institute Common Terminology Criteria for Adverse Events; TEAE, treatment-emergent adverse event; WBC, white blood cell. TEAEs presented by preferred term and most severe NCI-CTCAE grade*.

a *Grade ≥3 TEAEs reported in >5% of patients in the concurrent cohort, presented in descending order of incidence of all-grade TEAEs in the concurrent cohort*.

In this cohort, 21 patients (95.5%) who received any combination of *nab*-paclitaxel, carboplatin, or nivolumab had ≥1 TEAE that resulted in dose reduction and/or interruption, and 7 patients (31.8%) discontinued treatment due to TEAEs (Table 3a). The most common (incidence ≥10%) TEAEs leading to dose reduction and/or interruption were hematologic in nature. Two patients required nivolumab interruption due to pneumonitis. The most common TEAE leading to treatment discontinuation was decreased neutrophil count attributable to *nab*-paclitaxel. Results were generally similar in the nivolumab-treated subset (Supplementary Table 4A).

**Table 3a:** TEAEs leading to dose reduction, interruption, or discontinuation in the concurrent nivolumab cohort.

**Parameter, *n* (%)**	**Concurrent Cohort (*****n*** **=** **22)**			
	***nab*-Paclitaxel**	**Carboplatin**	**Nivolumab**	***nab*-Paclitaxel/carboplatin/nivolumab**
Patients with ≥1 TEAE leading to dose reduction or interruption^a^	20 (90.9)	14 (63.6)	14 (63.6)	21 (95.5)
Patients with ≥1 TEAE leading to withdrawal of study drug	4 (18.2)	1 (4.5)	4 (18.2)	7 (31.8)
Most common TEAEs leading to dose reduction and/or interruption^b^ Neutropenia	9 (40.9)	7 (31.8)	4 (18.2)	9 (40.9)
Neutrophil count decreased	6 (27.3)	3 (13.6)	1 (4.5)	6 (27.3)
Thrombocytopenia	5 (22.7)	3 (13.6)	2 (9.1)	5 (22.7)
Platelet count decreased	4 (18.2)	3 (13.6)	2 (9.1)	4 (18.2)
WBC count decreased	2 (9.1)	1 (4.5)	3 (13.6)	3 (13.6)
Fatigue	1 (4.5)	0	1 (4.5)	2 (9.1)
Anemia	2 (9.1)	1 (4.5)	1 (4.5)	2 (9.1)
ALT increased	1 (4.5)	1 (4.5)	2 (9.1)	2 (9.1)
Dehydration	2 (9.1)	0	2 (9.1)	2 (9.1)
Pneumonitis	0	0	2 (9.1)	2 (9.1)
Vomiting	1 (4.5)	1 (4.5)	2 (9.1)	2 (9.1)
Most common TEAEs leading to withdrawal of study drug^b^ Neutrophil count decreased	2 (9.1)	0	0	2 (9.1)

*ALT, alanine aminotransferase; TEAE, treatment-emergent adverse event; WBC, white blood cell*.

a *For nivolumab, because dose reductions were not allowed, the numbers represent patients with dose interruption*.

b *Occurring in >1 patient in any group, presented in descending order of incidence in the nab-paclitaxel/carboplatin/nivolumab group*.

Patients received treatment for a median of 32.25 weeks in a median of 9.0 cycles (see Supplementary Table 5 for additional treatment exposure and dose modification data, including those for the nivolumab-treated subset).

### Efficacy Outcomes (Concurrent Cohort; Dose-Finding and Expansion Parts)

**Figure 2:**
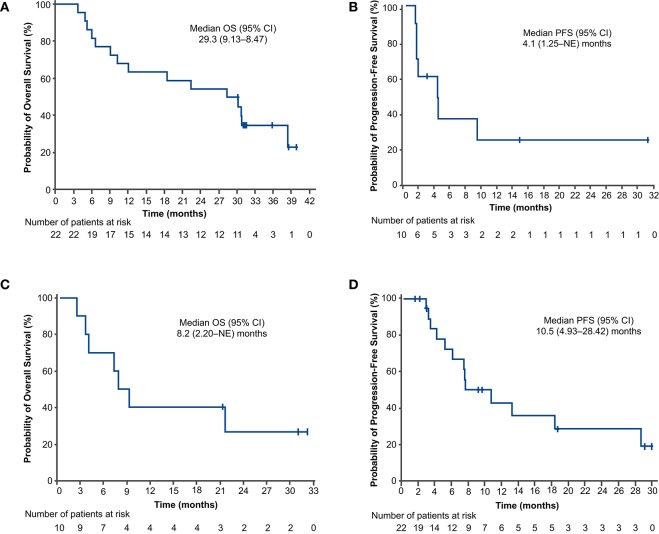
Progression-free survival **(A,C)** and overall survival **(B,D)** outcomes in the concurrent cohort (dose-finding and expansion parts; **A,B**) and delayed cohort (dose-finding part only; **C,D**). Two patients in the concurrent cohort and 4 patients in the delayed cohort did not receive nivolumab. CI, confidence interval; NE, not evaluable; OS, overall survival; PFS, progression-free survival.

**Figure 3:**
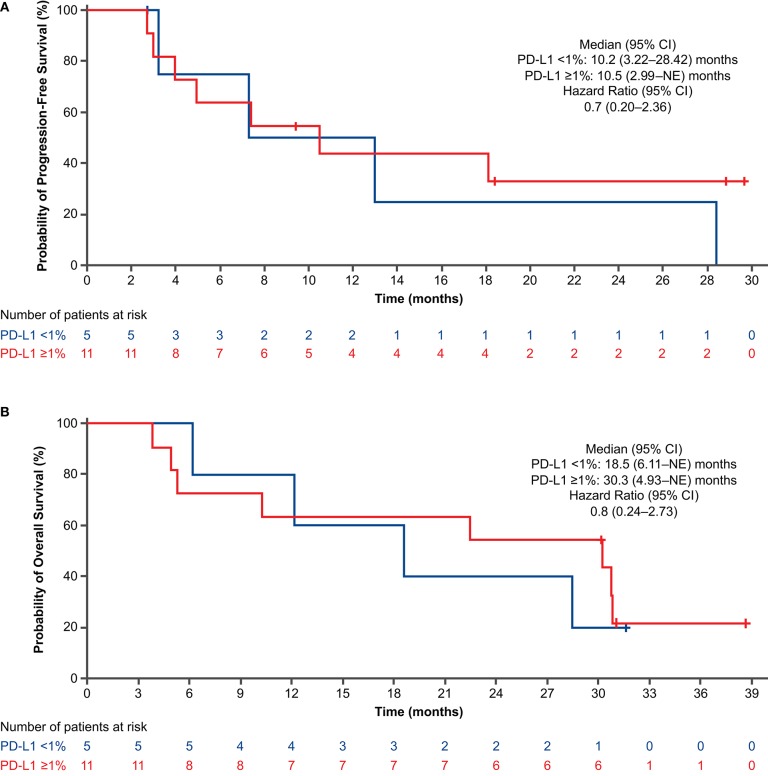
Progression-free survival **(A)** and overall survival **(B)** by PD-L1 expression in nivolumab-treated subset (concurrent cohort; dose-finding and expansion parts). CI, confidence interval; NE, not evaluable; PD-L1, programmed death ligand 1.

**Figure 4:**
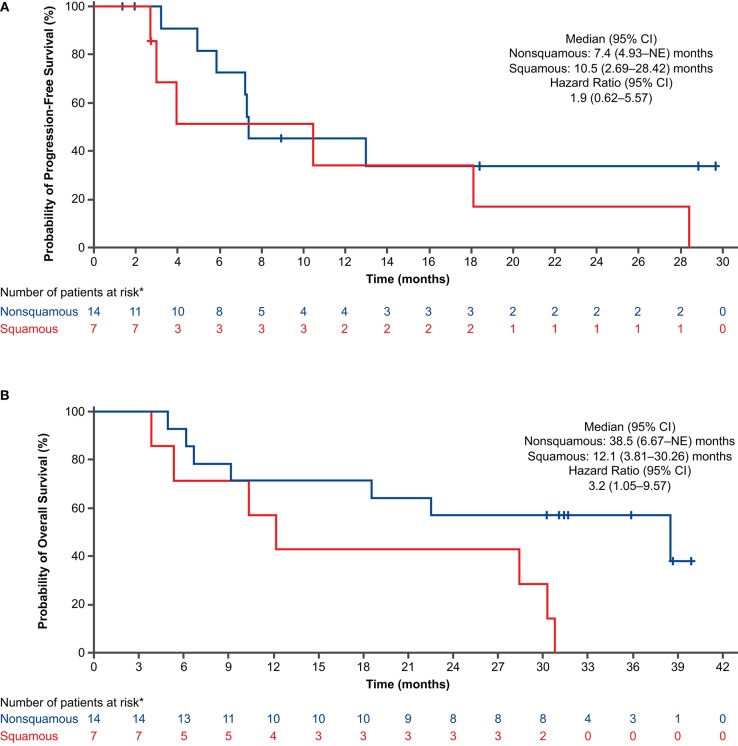
Progression-free survival **(A)** and overall survival **(B)** by histology in all treated patients (concurrent cohort; dose-finding and expansion parts). *Histology was not confirmed for 1 patient. CI, confidence interval; NE, not evaluable.

For the PFS analysis, 13 patients (59.1%) had disease progression or died. The investigator-assessed median PFS was 10.5 months (95% CI: 4.93–28.42) (Figure 2A), and the 1-year estimated PFS rate was 43.0% (95% CI: 19.76–64.43). The results were generally similar in the nivolumab-treated subset (Supplementary Table 6). Among patients with known baseline PD-L1 status (*n* = 16), the median PFS in the nivolumab-treated subset was similar in patients with PD-L1 expression <1% (10.2 months) and PD-L1 expression ≥1% (10.5 months) (Figure 3A). Among patients with confirmed histology (*n* = 21), the median PFS was 10.5 months in patients with squamous histology and 7.4 months in those with non-squamous histology (Figure 4A).

For the OS analysis, 15 patients (68.2%) had died. The median OS was 29.3 months (95% CI: 9.13–38.47) (Figure 2B), and the 1-year estimated OS rate was 68.2% (95% CI: 44.62–83.38). The results were similar in the nivolumab-treated subset (Supplementary Table 6). Among patients with known baseline PD-L1 status in the nivolumab-treated subset (*n* = 16), the median OS was numerically longer in patients with PD-L1 expression ≥1% (30.3 months) vs. PD-L1 expression <1% (18.5 months) (Figure 3B). Among patients with confirmed histology (*n* = 21), the median OS was numerically longer in patients with non-squamous (38.5 months) vs. squamous histology (12.1 months) (Figure 4B).

**Figure 5:**
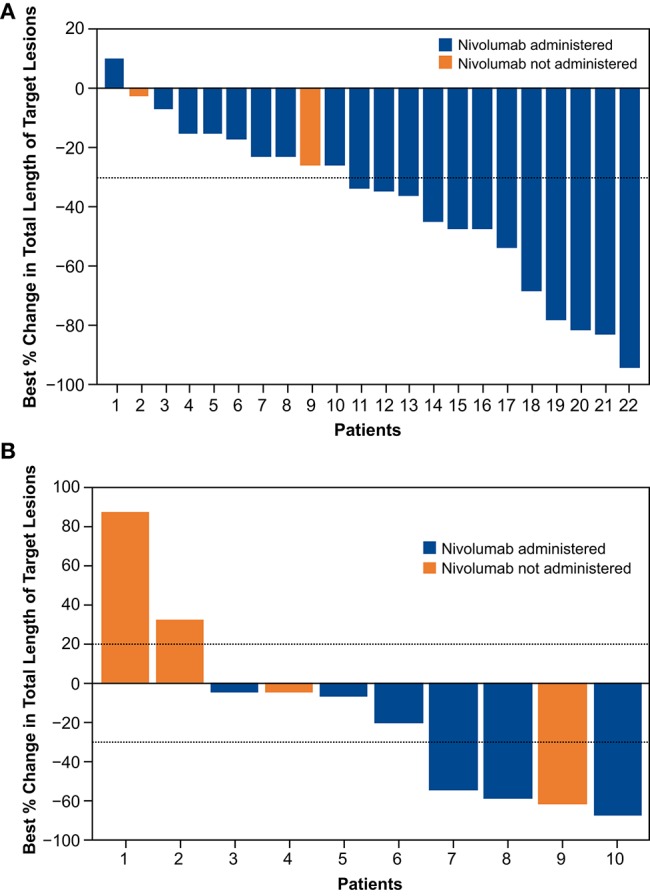
Change in total length of target lesions from baseline up to initial progression for individual patients in concurrent cohort (dose-finding and expansion parts; **A**) and delayed cohort (dose-finding part only; **B**).

The confirmed ORR was 45.5%, with 1 complete response and 9 partial responses (PRs) (Table 4); all responses occurred in nivolumab-treated patients. Among patients in the nivolumab-treated subset with available baseline PD-L1 expression levels (*n* = 16), the ORR was 40.0% in patients with PD-L1 expression <1% (PRs in 2 of 5 patients) and 63.6% in those with PD-L1 expression ≥1% [7 (1 complete response, 6 PRs) of 11 patients]. The DCR was 90.9%, and the median DOR was 9.2 months [95% CI: 3.25–not evaluable (NE)] (Table 4). The responses were generally similar in the nivolumab-treated population (Supplementary Table 7). The median best percent change from baseline in total length of target lesions was −35.1%; Figure 5A shows individual values. In this cohort, 4 patients were treated with nivolumab beyond the initial RECIST-defined progressive disease, and the best percent changes in total length of target lesions from the first disease progression event in these patients were −22, 0, 15, and 40%.

### Summary of Findings in the Delayed Cohort (Dose-Finding Part Only)

One DLT (grade 2 pneumonitis that did not resolve with dose delay and systemic steroids) was reported in the delayed cohort. All patients had a grade 3/4 TEAE, and 20.0% had ≥1 serious grade 3/4 TEAE (Table 2). Grade 3/4 TEAEs that occurred in ≥10% of patients were neutropenia, anemia, neutrophil count decreased, thrombocytopenia, and hypokalemia (Table 2). One patient (10%) experienced grade 3/4 peripheral neuropathy; grade 1/2 peripheral neuropathy was reported in 3 patients (30%). In this cohort, 8 patients (80.0%) who received any combination of *nab*-paclitaxel, carboplatin, or nivolumab had ≥1 TEAE that resulted in dose reduction and/or interruption, and 2 (20.0%) patients discontinued treatment due to TEAEs (Table 3b). The results were generally similar in the nivolumab-treated subset (Supplementary Table 4B).

**Table 3b:** TEAEs leading to dose reduction, interruption, or discontinuation in the delayed nivolumab cohort.

**Parameter, *n* (%)**	**Delayed cohort (dose-finding part only) (*****n*** **=** **10)**			
	***nab*-Paclitaxel**	**Carboplatin**	**Nivolumab**	***nab*-Paclitaxel/carboplatin/nivolumab**
Patients with ≥1 TEAE leading to dose reduction or interruption^a^	8 (80.0)	6 (60.0)	1 (10.0)	8 (80.0)
Patients with ≥1 TEAE leading to withdrawal of study drug	1 (10.0)	1 (10.0)	2 (20.0)	2 (20.0)
Most common TEAEs leading to dose reduction and/or interruption^b^ Neutropenia	4 (40.0)	2 (20.0)	0	4 (40.0)
Platelet count decreased	2 (20.0)	1 (10.0)	0	2 (20.0)
Fatigue	2 (20.0)	1 (10.0)	0	2 (20.0)
Most common TEAEs leading to withdrawal of study drug^c^ Myelopathy	0	0	1 (10.0)	1 (10.0)
Pneumonitis	0	0	1 (10.0)	1 (10.0)
Platelet count decreased	1 (10.0)	1 (10.0)	0	1 (10.0)

*TEAE, treatment-emergent adverse event*.

a *For nivolumab, because dose reductions were not allowed, the numbers represent patients with dose interruption*.

b *Occurring in >1 patient in any group, presented in descending order of incidence in the nab-paclitaxel/carboplatin/nivolumab group*.

c *Due to small numbers, all TEAEs leading to withdrawal are listed*.

**Table 4:** Response rates and duration of response.

**Parameter**	**Concurrent cohort** **(*n* = 22)**	**Delayed cohort (dose-finding part only) (*n* = 10)**
**Response rates**		
Best overall response up to initial progression, *n* (%)		
Confirmed complete response	1 (4.5)	0
Confirmed partial response	9 (40.9)	3 (30.0)
Stable disease ≥6 weeks	10 (45.5)	3 (30.0)
Progressive disease	1 (4.5)	4 (40.0)
Not evaluable	1 (4.5)	0
Confirmed overall response rate, *n* (% [95% CI])	10 (45.5 [24.4–67.8])	3 (30.0 [6.7–65.2])
Disease control rate, *n* (% [95% CI])	20 (90.9 [70.8–98.9])	6 (60.0 [26.2–87.8])
**Duration of response**^a^		
Patients who subsequently had progressive disease or died, *n* (%)	6 (60.0)	NR
Median (95% CI), months	9.2 (3.25–NE)	NR

*CI, confidence interval; NE, not evaluable; NR, not reported*.

a *Only patients who had confirmed complete or partial response are included (n = 10)*.

Patients in the delayed cohort received treatment for a median of 17.55 weeks in a median of 4.5 cycles (see Supplementary Table 5 for additional treatment exposure and dose modification data, including those for the nivolumab-treated subset).

For the PFS analysis, 7 patients (70.0%) in the delayed cohort had disease progression or died. The investigator-assessed median PFS was 4.1 months (95% CI: 1.25–NE; Figure 2C), and the 1-year estimated PFS rate was 24.0% (95% CI: 3.77–53.73). In patients with non-squamous histology, the investigator-assessed median PFS was 2.9 months; it was not evaluable in patients with squamous histology. The investigator-assessed median PFS was not evaluable in the nivolumab-treated subset of patients with PD-L1 expression <1%; in those with PD-L1 expression ≥1%, it was 4.1 months.

For the OS analysis, 7 patients (70.0%) had died. The median OS was 8.2 months (95% CI: 2.20–NE; Figure 2D), and the 1-year estimated OS rate was 40.0% (95% CI: 12.27–67.02). Overall survival results for the nivolumab-treated subset are summarized in Supplementary Table 6. In patients with non-squamous histology, the median OS was 7.3 months; it was not evaluable in patients with squamous histology. The median OS was not evaluable in the nivolumab-treated patients with PD-L1 expression <1%; in those with PD-L1 expression ≥1%, it was 7.6 months.

As noted in the “Patients and Treatment” section, 4 of the 10 patients assigned to the delayed cohort discontinued treatment before cycle 3. The PFS and OS outcomes in the nivolumab-treated subset of patients in the delayed cohort (Supplementary Table 6) were markedly different from those in all patients in the delayed cohort described above in this section.

The ORR was 30.0%; 3 patients—all of whom received nivolumab—had a confirmed PR; 2 of the 3 PRs were noted before nivolumab administration. Among patients with PD-L1 data available (6 patients: 5 with PD-L1 <1% and 1 with PD-L1 ≥1%), the ORR was 60.0% in nivolumab-treated patients with PD-L1 expression <1% (PRs in 3 of 5 patients); no response was observed in the patient with PD-L1 expression ≥1%. The DCR was 60.0%, and the median best percent change from baseline in total length of target lesions was −13.1%; Figure 5B shows individual values. In this cohort, 2 patients were treated with nivolumab beyond the initial RECIST-defined progressive disease, and the best percent changes in total length of target lesions from the first disease progression event in these patients were 0 and 16%.

## Discussion

This trial demonstrated the feasibility of concurrent administration of nivolumab with *nab*-paclitaxel/carboplatin in cycle 1 for the treatment of advanced NSCLC. No DLTs were reported in the concurrent cohort, and 1 DLT was reported in the delayed cohort; no new safety signals were identified in either cohort. Although preliminary and based on a relatively small sample size, the efficacy data with *nab*-paclitaxel and carboplatin plus nivolumab in the concurrent cohort (median PFS, 10.5 months; median OS, 29.3 months; DCR, 90.9%) were generally encouraging.

Several phase III studies have recently reported results of first-line treatment of advanced NSCLC with immunotherapy and platinum chemotherapy-based combinations (4–8, 14). In these studies, the safety profile with chemotherapy plus immunotherapy was reported to be generally consistent with the known safety profile of the individual agents. The present study did not allow for comparison of safety between chemotherapy plus nivolumab and chemotherapy alone because no chemotherapy-alone cohort was included; however, the safety profile was as expected and generally similar in the concurrent and delayed cohorts.

The delayed cohort was not studied beyond the dose-finding part because the efficacy outcome results were not as encouraging as those in the concurrent cohort and the safety data supported concurrent administration of nivolumab with *nab*-paclitaxel/carboplatin. In the delayed cohort, 4 of 10 patients discontinued before cycle 3 and, therefore, never received nivolumab. Among the patients in the delayed cohort who remained on treatment long enough to receive nivolumab in cycle 3, the median PFS and OS values, although numerically lower, were closer to those in the patients who initiated nivolumab in cycle 1. In addition to treatment discontinuation early in the course of therapy, differences in efficacy between the 2 cohorts could possibly be explained by demographics (age or sex), baseline clinical characteristics (ECOG PS or PD-L1 status), or treatment exposure, as well as timing of nivolumab initiation.

Although the data are limited, they suggest that delaying immune checkpoint inhibitors is suboptimal due to the greater potential for early disease progression; this reinforces the importance of delivering all agents in cycle 1 to provide the best opportunity for patient benefit. This is further supported by the short median time to response of 1.4 months reported for the concurrent regimen in KEYNOTE-407 (5).

In summary, this study and others have demonstrated that *nab*-paclitaxel/carboplatin can be safely combined when administered concurrently with immune checkpoint inhibitors—nivolumab, atezolizumab, or pembrolizumab—in advanced NSCLC. The composite safety data, along with efficacy data from randomized, phase III trials, support the rationale for *nab*-paclitaxel/carboplatin as a backbone chemotherapy regimen in combination with immunotherapy for advanced NSCLC.

## Data Availability Statement

Data requests may be submitted to Celgene at www.CelgeneClinicalDataSharing.com and must include a description of the research proposal.

## Ethics Statement

The studies involving human participants were reviewed and approved by institutional review board or independent ethics committee at each study site. The patients provided their written informed consent to participate in this study.

## Author Contributions

All authors were involved with data interpretation and critically revising the report, reviewed and approved the final version of the report to submit for publication, agreed to be accountable for all aspects of the work, and to ensure that questions related to the accuracy or integrity of any part of the work are appropriately investigated and resolved.

### Conflict of Interest

JG has received research grants from Bristol-Myers Squibb, Roche/Genentech, AstraZeneca/MedImmune, and Merck and has served as a speaker for Merck. DW has served in advisory roles for AbbVie, Amgen, AstraZeneca, Bristol-Myers Squibb, Celgene, and Janssen and has served as a consultant for CTI Biopharma and McGivney Global and as a speaker for Bristol-Myers Squibb, Celgene, Janssen, Roche/Genentech, and Lilly. BG has served in consulting/advisory roles for Celgene, Cook Medical, Foundation Medicine, Ipsen, Merrimack, Exelixis, Taiho Oncology, Bristol-Myers Squibb, and Terumo Interventional Systems. PO'D has received research support from Pfizer, Genentech, Bristol-Myers Squibb, GlaxoSmithKline, Five Prime, Forty Seven, Boston Biomedical Inc., Novartis, Celgene, Incyte, Lilly/ImClone, Array, H3 Biomedicine, and Taiho; has served as a consultant for Genentech and Celgene; and has given expert testimony for Bayer and Lilly. RB was an employee of Celgene at the time of conduct of the study. SB, LL, CL, and TO are employees of Celgene and hold stock options. KK has served in advisory roles for Bristol-Myers Squibb.

## Abbreviations

AE: adverse event
ALK: anaplastic lymphoma kinase
ALT: alanine aminotransferase
AUC: area under the curve
CI: confidence interval
DCR: disease control rate
DLT: dose-limiting toxicity
ECOG PS: Eastern Cooperative Oncology Group performance status
EGFR: epidermal growth factor receptor
NCI-CTCAE: National Cancer Institute's Common Terminology Criteria for Adverse Events
NE: not evaluable
NR: not reported
NSCLC: non-small cell lung cancer
ORR: overall response rate
OS: overall survival
PD-L1: programmed death ligand 1
PFS: progression-free survival
PR: partial response
RECIST: Response Evaluation Criteria in Solid Tumors
RP2D: recommended part 2 dose
TEAE: treatment-emergent adverse event
WBC: white blood cell.
