# Supplementary material for: Safety and Efficacy Results of a Phase I, Open-Label Study of Concurrent and Delayed Nivolumab in Combination With nab-Paclitaxel and Carboplatin in Advanced Non-small Cell Lung Cancer
Source: Front Oncol. 2019 Nov 26;9:1256. doi: 10.3389/fonc.2019.01256 (PMC6901975; doi:10.3389/fonc.2019.01256)
Supplement: Supplementary file 1 [file Table_1.DOCX]

***Supplementary Material***

Table of Contents

[1 Supplementary Methods 2](#_Toc17186203)

[1.1 DLT-evaluable population 2](#_Toc17186204)

[1.2 DLT definition 2](#_Toc17186205)

[1.3 Nivolumab dose details 2](#_Toc17186206)

[1.4 Dose modifications 2](#_Toc17186207)

[1.5 Eligibility criteria for nivolumab treatment beyond disease progression 3](#_Toc17186208)

[Supplementary Table 1. List of institutional review board sites 4](#_Toc17186209)

[Supplementary Table 2. Demographic and baseline clinical characteristics (nivolumab-treated subset) 6](#_Toc17186210)

[Supplementary Table 3. Safety outcomes, including select TEAEs of special interest attributable to nivolumab (nivolumab-treated subset) 8](#_Toc17186211)

[Supplementary Table 4A. TEAEs leading to dose reduction, delay, or discontinuation in the concurrent cohort (nivolumab-treated subset) 9](#_Toc17186212)

[Supplementary Table 4B. TEAEs leading to dose reduction, delay, or discontinuation in the delayed cohort (nivolumab-treated subset) 10](#_Toc17186213)

[Supplementary Table 5. Treatment exposure and dose modifications 11](#_Toc17186214)

[Supplementary Table 6. Time-to-event analyses (nivolumab-treated subset) 13](#_Toc17186215)

[Supplementary Table 7. Response rates and duration of response (nivolumab-treated subset) 14](#_Toc17186216)

# 1 Supplementary Methods

## 1.1 DLT-evaluable population

The dose-limiting toxicity (DLT)–evaluable population comprised all patients enrolled in the dose-finding part who received ≥2 cycles of all study drugs, including nivolumab at the assigned dose, and remained in the study for 14 days after the last nivolumab dose in the second cycle, those who received ≥1 dose of nivolumab and discontinued treatment due to a DLT before completing the full second cycle, or those who had an equivocal DLT after receiving ≥1 dose of nivolumab and deemed by the safety oversight committee as DLT evaluable.

## 1.2 DLT definition

A DLT was defined as a treatment-related adverse event (AE) occurring during the first 2 cycles that met ≥1 of the following criteria: grade ≥2 uveitis or eye pain that did not improve with topical treatment to grade 1 within ≤6 weeks and/or prior to next nivolumab dose or required systemic treatment; grade ≥2 pneumonitis or interstitial lung disease that did not resolve with dose delay and systemic steroids; grade ≥3 febrile neutropenia; grade 3 nonskin AE regardless of duration (except laboratory abnormalities); grade ≥4 AEs, including laboratory abnormalities (except grade 4 neutropenia or leukopenia without fever that lasted <7 days); grade 3 thrombocytopenia associated with bleeding; and specific liver function laboratory abnormalities (alanine aminotransferase [ALT] or aspartate aminotransferase [AST] levels >8 × upper limit of normal [ULN]; total bilirubin levels >5 × ULN; ALT/AST levels >5 to 8 × ULN and/or total bilirubin levels >3 × ULN for 2 weeks; or ALT/AST levels >3 × ULN and total bilirubin >2 × ULN with alkaline phosphatase activity <2 × ULN with no explanation for the combined elevations).

## 1.3 Nivolumab dose details

Nivolumab dose level was decided based on DLTs in the first 2 cycles; starting at the 5-mg/kg dose level, nivolumab dose level could be sequentially de-escalated to 3 and 1 mg/kg, if DLTs occurred. Assessment of ≥6 DLT-evaluable patients was required to declare a dose level safe, but dose could be de-escalated with assessment of ≥2 patients. If ≤1 DLT occurred in the first 6 patients at a given dose level, and the dose level was deemed safe by the safety oversight committee, this dose was to become the recommended part 2 dose (RP2D) and the study advanced to the expansion part. If deemed safe, treatment arms could be expanded using the RP2D. If >1 DLT occurred at a dose level, and the dose level was deemed unsafe, the dose of nivolumab was to be de-escalated. A treatment arm could not have proceeded to the expansion part if >1 DLT occurred even at the lowest dose level (1 mg/kg).

## 1.4 Dose modifications

Once the safe dose level was established, nivolumab dose could not be reduced for individual patients, but it could be modified. Nivolumab dose modifications (delays/interruptions or discontinuations) were based on prespecified laboratory and AE criteria. Generally, for immune-related AEs (irAEs) with no or mild symptoms (grade 1), nivolumab was continued per protocol; for irAEs with moderate symptoms (typically grade 2), nivolumab therapy was delayed/interrupted and resumed if symptoms improved with corticosteroids; and for irAEs with severe symptoms (typically grade 3/4), nivolumab was discontinued. Nivolumab was also discontinued if it was interrupted for >6 weeks, with a few exceptions. Doses of *nab*-paclitaxel and carboplatin could be adjusted according to their package inserts.

## 1.5 Eligibility criteria for nivolumab treatment beyond disease progression

Patients could continue nivolumab treatment beyond initial disease progressive (Response Evaluation Criteria in Solid Tumors version 1.1) if they continued to meet all other eligibility criteria, had stable performance status, exhibited tolerance to nivolumab, and were assessed by investigator to have achieved clinical benefit without rapid disease progression or clinical deterioration, and if the treatment continuation did not delay any imminent intervention to prevent serious complications of disease progression.

# Supplementary Table 1. List of institutional review board sites

| **Site No.** | **Name/Address of IEC/IRB** |
| --- | --- |
| 001 | Medical College of Wisconsin / Froedtert Hospital Institutional Review Board – Human Research Protection Program  8701 Watertown Plank Rd HRC- MACC FUND 3040  Milwaukee, WI 53226 USA |
| 002 | Western Institutional Review Board  1019 39th Ave SE, Suite 120  Puyallup, WA 98374 USA |
| 004 | Western Institutional Review Board  1019 39th Ave SE, Suite 120  Puyallup, WA 98374 USA |
| 005 | Western Institutional Review Board  1019 39th Ave SE, Suite 120  Puyallup, WA 98374 USA |
| 006 | Office of the Human Research Protection Program (OHRPP)  10889 Wilshire Blvd, Suite 830  Los Angeles, CA 90095 USA  Previous:  Office of the Human Research Protection Program (OHRPP)  11000 Kinross Avenue, Suite 211  Los Angeles, CA 90095 USA |
| 008 | IntegReview Ethical Review Board  3001 S. Lamar Boulevard, Suite 210  Austin, TX 78704 USA |
| 009 | Chesapeake Research Review, Inc.  6940 Columbus Gateway Drive, Suite 110  Columbia, MD 21046 USA |
| 010 | Western Institutional Review Board  1019 39th Ave SE, Suite 120  Puyallup, WA 98374 USA |
| 011 | Chesapeake Research Review, Inc.  6940 Columbus Gateway Drive, Suite 110  Columbia, MD 21046 USA  Previous:  Liberty IRB  1450 S Woodland, Suite 300A  DeLand, FL 32720 USA |
| 012 | Dana Farber Cancer Institute IRB  450 Brookline Avenue, OS229  Boston, MA 02215 USA |
| 014 | Institutional Review Board, University of California, Davis  CTSC Building, 2921 Stockton Blvd, Suite 1400, Room 1429  Sacramento, CA 95817 USA |
| 015 | University of Pennsylvania Office of Regulatory Affairs  3800 Spruce Street, First Floor Room 151  Philadelphia, PA 19104 USA  Previous:  University of Pennsylvania Office of Regulatory Affairs  3624 Market Street, Suite 301 South  Philadelphia, PA 19104 USA |
| 016 | Yale University Institutional Review Board #2, 3, 4B, 5 – Human Investigation Committee I, II, III, IV  150 Munson Street, 3rd Floor  New Haven, CT 06520 USA |
| IEC, independent ethics committee; IRB, institutional review board. | |

# **Supplementary Table 2.** Demographic and baseline clinical characteristics (nivolumab-treated subset)

| **Characteristic** | **Concurrent Cohort (n = 20)** | **Delayed Cohort  (dose-finding part only)  (n = 6)** | |
| --- | --- | --- | --- |
| Age, median (range), years  <65 years, n (%)  ≥65 years, n (%) | 65.5 (38–77)  9 (45.0)  11 (55.0) | 70.0 (44–82)  2 (33.3)  4 (66.7) |  |
| Sex, n (%)  Male  Female | 5 (25.0)  15 (75.0) | 4 (66.7)  2 (33.3) |  |
| Race, n (%)  White  Asian  Black or African American  Not collected or reported | 16 (80.0)  1 (5.0)  0  3 (15.0) | 5 (83.3)  0  1 (16.7)  0 |  |
| ECOG PS, n (%)  0  1 | 6 (30.0)  14 (70.0) | 5 (83.3)  1 (16.7) |  |
| Stage at primary diagnosis, n (%)  IA  IB  IIB  IIIA  IIIB  IVA  IVB  Unknown | 2 (10.0)  1 (5.0)  0  2 (10.0)  1 (5.0)  10 (50.0)  2 (10.0)  2 (10.0) | 0  0  1 (16.7)  1 (16.7)  1 (16.7)  2 (33.3)  1 (16.7)  0 |  |
| Histology, n (%)  Confirmed  Adenocarcinoma  Squamous cell carcinoma  Large cell carcinoma  Other  Not confirmed | 19 (95.0)  10 (50.0)  7 (35.0)  0  2 (10.0)  1 (5.0) | 6 (100.0)  4 (66.7)  2 (33.3)  0  0  0 |  |
| PD-L1 category, n (%)  <1%  ≥1%  Missing | 5 (25.0)  11 (55.0)  4 (20.0) | 5 (83.3)  1 (16.7)  0 |  |
| *KRAS* status, n (%)  *KRAS* mutant  *KRAS* wild type  Unknown | 2 (10.0)  3 (15.0)  15 (75.0) | 1 (16.7)  1 (16.7)  4 (66.7) |  |
| *ALK* status, n (%)  *ALK* wild type  Unknown | 7 (35.0)  13 (65.0) | 3 (50.0)  3 (50.0) |  |
| *EGFR* status, n (%)  *EGFR* mutant  *EGFR* wild type  Unknown | 2 (10.0)  6 (30.0)  12 (60.0) | 0  2 (33.3)  4 (66.7) | |
| Prior anticancer therapy, n (%) |  |  | |
| Systemic therapy | 5 (25.0) | 1 (16.7) | |
| Radiation | 8 (40.0) | 1 (16.7) | |
| Surgery | 4 (20.0) | 4 (66.7) | |

ALK, anaplastic lymphoma kinase; ECOG PS, Eastern Cooperative Oncology Group performance status; EGFR, epidermal growth factor receptor; PD-L1, programmed death ligand 1.

# **Supplementary Table 3.** Safety outcomes, including select TEAEs of special interest attributable to nivolumab (nivolumab-treated subset)

| **Parameter, n (%)** | **Concurrent Cohort**  **(n = 20)** | | **Delayed Cohort**  **(dose-finding part only)**  **(n = 6)** | |
| --- | --- | --- | --- | --- |
|  | **All Grade** | **Grade 3/4** | **All Grade** | **Grade 3/4** |
| Patients with ≥1 TEAE | 20 (100.0) | 18 (90.0) | 6 (100.0) | 6 (100.0) |
| Patients with ≥1 serious TEAE | 8 (40.0) | 6 (30.0) | 2 (33.3) | 1 (16.7) |
| Patients with ≥1 TEAE of special interest attributable to nivolumab | 16 (80.0) | 3 (15.0) | 5 (83.3) | 2 (33.3) |
| Most common TEAEs^a^ |  |  |  |  |
| Vomiting | 12 (60.0) | 3 (15.0) | 1 (16.7) | 0 |
| Neutropenia | 11 (55.0) | 9 (45.0) | 2 (33.3) | 2 (33.3) |
| Anemia | 11 (55.0) | 8 (40.0) | 5 (83.3) | 2 (33.3) |
| Thrombocytopenia | 7 (35.0) | 2 (10.0) | 1 (16.7) | 0 |
| Hypokalemia | 5 (25.0) | 3 (15.0) | 1 (16.7) | 1 (16.7) |
| Neutrophil count decreased | 4 (20.0) | 4 (20.0) | 3 (50.0) | 2 (33.3) |
| WBC decreased | 4 (20.0) | 2 (10.0) | 1 (16.7) | 0 |
| Pneumonia | 3 (15.0) | 2 (10.0) | – | – |
| Hyponatremia | 2 (10.0) | 2 (10.0) | 1 (16.7) | 0 |
| Select TEAEs of special interest attributable to nivolumab, n (%)^b^ |  |  |  |  |
| Hypothyroidism | 3 (15.0) | 0 | – | – |
| Pneumonitis | 2 (10.0) | 0 | 1 (16.7) | 0 |

NCI-CTCAE, National Cancer Institute Common Terminology Criteria for Adverse Events; TEAE, treatment-emergent adverse event; WBC, white blood cell. TEAEs presented by preferred term and most severe NCI-CTCAE grade.

^a^ Grade ≥3 TEAEs reported in >5% of patients in the concurrent cohort, presented in descending order of incidence of all-grade TEAEs in the concurrent cohort.

^b^ All-grade select TEAEs of special interest reported in ≥2 patients in the concurrent cohort.

# **Supplementary** Table 4A. TEAEs leading to dose reduction, delay, or discontinuation in the concurrent cohort (nivolumab-treated subset)

| **Parameter, n (%)** | **Concurrent Cohort (dose-finding and expansion parts) (n = 20)** | | | |
| --- | --- | --- | --- | --- |
|  | ***nab*-Paclitaxel** | **Carboplatin** | **Nivolumab** | ***nab*-Paclitaxel/ Carboplatin/Nivolumab** |
| Patients with ≥1 TEAE leading to dose reduction or interruption^a^ | 18 (90.0) | 13 (65.0) | 13 (65.0) | 19 (95.0) |
| Patients with ≥1 TEAE leading to withdrawal of study drug^b^ | 2 (10.0) | 1 (5.0) | 4 (20.0) | 5 (25.0) |
| TEAEs leading to dose reduction and/or interruption^c^ |  |  |  |  |
| Neutropenia | 9 (45.0) | 7 (35.0) | 4 (20.0) | 9 (45.0) |
| Thrombocytopenia | 5 (25.0) | 3 (15.0) | 2 (10.0) | 5 (25.0) |
| Platelet count decreased | 4 (20.0) | 3 (15.0) | 2 (10.0) | 4 (20.0) |
| Neutrophil count decreased | 4 (20.0) | 2 (10.0) | 1 (5.0) | 4 (20.0) |
| WBC count decreased | 1 (5.0) | 0 | 2 (10.0) | 2 (10.0) |
| ALT increased | 1 (5.0) | 1 (5.0) | 2 (10.0) | 2 (10.0) |
| Anemia | 2 (10.0) | 1 (5.0) | 1 (5.0) | 2 (10.0) |
| Dehydration | 2 (10.0) | 0 | 2 (10.0) | 2 (10.0) |
| Fatigue | 1 (5.0) | 0 | 1 (5.0) | 2 (10.0) |
| Pneumonitis | 0 | 0 | 2 (10.0) | 2 (10.0) |
| Vomiting | 1 (5.0) | 1 (5.0) | 2 (10.0) | 2 (10.0) |

ALT, alanine aminotransferase; TEAE, treatment-emergent adverse event; WBC, white blood cell.

^a^ For nivolumab, because dose reductions were not allowed, the numbers represent patients with dose interruption.

^b^ None of the TEAEs leading to withdrawal of study drug were reported in >1 patient in any group of any cohort.

^c^ Occurring in >1 patient in any group, presented in descending order of incidence in the *nab*-paclitaxel/carboplatin/nivolumab group.

# Supplementary Table 4B. TEAEs leading to dose reduction, delay, or discontinuation in the delayed cohort (nivolumab-treated subset)

| **Parameter, n (%)** | **Delayed Cohort  (dose-finding part only)**  **(n = 6)** | | | |
| --- | --- | --- | --- | --- |
|  | ***nab*-Paclitaxel** | **Carboplatin** | **Nivolumab** | ***nab*-Paclitaxel/ Carboplatin/Nivolumab** |
| Patients with ≥1 TEAE leading to dose reduction or interruption^a^ | 5 (83.3) | 5 (83.3) | 1 (16.7) | 5 (83.3) |
| Patients with ≥1 TEAE leading to withdrawal of study drug^b^ | 1 (16.7) | 1 (16.7) | 2 (33.3) | 2 (33.3) |
| TEAEs leading to dose reduction and/or interruption^c^ |  |  |  |  |
| Neutropenia | 2 (33.3) | 2 (33.3) | 0 | 2 (33.3) |
| Platelet count decreased | 2 (33.3) | 1 (16.7) | 0 | 2 (33.3) |

TEAE, treatment-emergent adverse event.

^a^ For nivolumab, because dose reductions were not allowed, the numbers represent patients with dose interruption.

^b^ None of the TEAEs leading to withdrawal of study drug were reported in >1 patient in any group of any cohort.

^c^ Occurring in >1 patient in any group.

# **Supplementary Table 5.** Treatment exposure and dose modifications

| **Parameter** | **Concurrent Cohort** | | **Delayed Cohort  (dose-finding part only)** | |
| --- | --- | --- | --- | --- |
|  | **All Patients  (n = 22)** | **Nivolumab-Treated Subset (n = 20)** | **All Patients (n = 10)** | **Nivolumab-Treated Subset (n = 6)** |
| **Treatment exposure** | | | | |
| Treatment duration, median (range), weeks | 32.25 (4.1–127.9) | 36.70 (4.1–127.9) | 17.55 (6.1–138.9) | 43.75 (16.1–138.9) |
| Treatment cycles, median (range), n | 9.0 (1–38) | 9.5 (1–38) | 4.5 (2–45) | 12.5 (4–45) |
| Relative dose intensity, median (range), %  *nab*-Paclitaxel  Carboplatin  Nivolumab | 66.90 (39.3–100.0)  75.34 (39.3–100.0)  84.62 (24.7–100.3)^a^ | 69.81 (39.3–100.0)  75.34 (39.3–100.0)  84.62 (24.7–100.3) | 60.35 (38.3–100.0)  81.19 (50.0–100.0)  94.84 (29.6–99.8)^a^ | 58.04 (38.3–100.0)  78.10 (50.0–100.0)  94.84 (29.6–99.8) |
| Dose intensity, median (range)  *nab*-Paclitaxel, mg/m^2^/week  Carboplatin, AUC/week  Nivolumab, mg/kg/week | 66.90 (39.3–100.0)  1.51 (0.8–2.0)  1.41 (0.4–1.7)^a^ | 69.81 (39.3–100.0)  1.51 (0.8–2.0)  1.41 (0.4–1.7) | 60.35 (38.3–100.0)  1.62 (1.0–2.0)  1.58 (0.5–1.7)^a^ | 58.04 (38.3–100.0)  1.56 (1.0–2.0)  1.58 (0.5–1.7) |
| Cumulative dose, median (range)  *nab*-Paclitaxel, mg/m^2^  Carboplatin, AUC  Nivolumab, mg/kg | 912.5 (275–1200)  21.8 (6–24)  47.5 (5–190)^a^ | 937.5 (300–1200)  22.5 (6–24)  47.5 (5–190) | 575.0 (375–1200)  16.5 (11–24)  52.5 (5–215)^a^ | 812.5 (575–1200)  21.0 (15–24)  52.5 (5–215) |
| **Dose modifications** | | | | |
| Patients with ≥1 dose reduction, n (%)^b^  *nab*-Paclitaxel  Carboplatin  Nivolumab | 17 (77.3)  14 (63.6)  0 | 15 (75.0)  13 (65.0)  0 | 7 (70.0)  6 (60.0)  0 | 5 (83.3)  5 (83.3)  0 |
| Patients with ≥1 dose delay, n (%)  *nab*-Paclitaxel  Carboplatin  Nivolumab | 14 (63.6)  13 (59.1)  13 (59.1) | 14 (70.0)  13 (65.0)  13 (65.0) | 7 (70.0)  6 (60.0)  3 (30.0) | 5 (83.3)  4 (66.7)  3 (50.0) |
| Patients with ≥1 dose not administered, n (%)  *nab*-Paclitaxel  Carboplatin  Nivolumab | 18 (81.8)  2 (9.1)  14 (63.6) | 16 (80.0)  2 (10.0)  12 (60.0) | 8 (80.0)  1 (10.0)  3 (30.0) | 4 (66.7)  1 (16.7)  3 (50.0) |

AUC, area under the curve.

^a^ Patients who did not receive nivolumab were not included in the summary of nivolumab exposure.

^b^ Per protocol, nivolumab dose reduction was not permitted for individual patients.

# Supplementary Table 6. Time-to-event analyses (nivolumab-treated subset)

| **Outcome** | **Concurrent Cohort  (n = 20)** | **Delayed Cohort  (n = 6)** |
| --- | --- | --- |
| **Progression-free survival**  Patients who had progressive disease or died, n (%)  Median (95% CI), months  1-year rate (95% CI), % | 13 (65.0)  10.5 (4.93–28.42)  43.0 (19.76–64.43) | 3 (50.0)  9.2 (4.11–NE)  40.0 (5.20–75.28) |
| **Overall survival**  Patients who died, n (%)  Median (95% CI), months  1-year rate (95% CI), % | 14 (70.0)  25.4 (6.67–30.85)  65.0 (40.30–81.53) | 3 (50.0)  21.3 (7.56–NE)  66.7 (19.46–90.44) |

CI, confidence interval; NE, not evaluable.

# Supplementary Table 7. Response rates and duration of response (nivolumab-treated subset)

| **Parameter** | **Concurrent Cohort  (n = 20)** | **Delayed Cohort**  **(n = 6)** |
| --- | --- | --- |
| **Response rates** |  |  |
| Best overall response up to initial progression, n (%) |  |  |
| Confirmed complete response | 1 (5.0) | 0 |
| Confirmed partial response | 9 (45.0) | 3 (50.0) |
| Stable disease ≥6 weeks | 8 (40.0) | 3 (50.0) |
| Progressive disease | 1 (5.0) | 0 |
| Not evaluable | 1 (5.0) | 0 |
| Confirmed overall response rate, n (%) | 10 (50.0) | 3 (50.0) |
| Disease control rate, n (%) | 18 (90.0) | 6 (100.0) |
| **Duration of response^a^** |  |  |
| Patients who subsequently had progressive disease or died, n (%) | 6 (60.0) | NR |
| Median (95% CI), months | 9.2 (3.25–NE) | NR |

CI, confidence interval; NE, not evaluable; NR, not reported.

^a^ Only patients who had confirmed complete or partial response are included (n = 10).
